# Radiogenomic Analysis of Papillary Thyroid Carcinoma for Prediction of Cervical Lymph Node Metastasis: A Preliminary Study

**DOI:** 10.3389/fonc.2021.682998

**Published:** 2021-06-29

**Authors:** Yuyang Tong, Peixuan Sun, Juanjuan Yong, Hongbo Zhang, Yunxia Huang, Yi Guo, Jinhua Yu, Shichong Zhou, Yulong Wang, Yu Wang, Qinghai Ji, Yuanyuan Wang, Cai Chang

**Affiliations:** ^1^ Department of Ultrasound, Fudan University Shanghai Cancer Center, Shanghai, China; ^2^ Department of Oncology, Shanghai Medical College, Fudan University, Shanghai, China; ^3^ Department of Surgical Oncology, The Ohio State University, Columbus, OH, United States; ^4^ Diagnostic Imaging Center, Shanghai Children’s Medical Center, Shanghai Jiao Tong University School of Medicine, Shanghai, China; ^5^ Department of Pathology, Sun Yat-sen Memorial Hospital, Sun Yat-sen University, Guangzhou, China; ^6^ Pharmaceutical Sciences Laboratory, Åbo Akademi University, Turku, Finland; ^7^ Turku Biosciences Center, University of Turku and Åbo Akademi University, Turku, Finland; ^8^ Department of Electronic Engineering, Fudan University and Key Laboratory of Medical Imaging Computing and Computer Assisted Intervention of Shanghai, Shanghai, China; ^9^ Department of Head and Neck Surgery, Fudan University Shanghai Cancer Center, Shanghai, China

**Keywords:** radiogenomic, papillary thyroid carcinoma, cervical lymph node metastasis, ultrasound, radiomics

## Abstract

**Background:**

Papillary thyroid carcinoma (PTC) is characterized by frequent metastases to cervical lymph nodes (CLNs), and the presence of lymph node metastasis at diagnosis has a significant impact on the surgical approach. Therefore, we established a radiomic signature to predict the CLN status of PTC patients using preoperative thyroid ultrasound, and investigated the association between the radiomic features and underlying molecular characteristics of PTC tumors.

**Methods:**

In total, 270 patients were enrolled in this prospective study, and radiomic features were extracted according to multiple guidelines. A radiomic signature was built with selected features in the training cohort and validated in the validation cohort. The total protein extracted from tumor samples was analyzed with LC/MS and iTRAQ technology. Gene modules acquired by clustering were chosen for their diagnostic significance. A radiogenomic map linking radiomic features to gene modules was constructed with the Spearman correlation matrix. Genes in modules related to metastasis were extracted for Gene Ontology (GO) and Kyoto Encyclopedia of Genes and Genomes (KEGG) pathway enrichment analyses, and a protein-protein interaction (PPI) network was built to identify the hub genes in the modules. Finally, the screened hub genes were validated by immunohistochemistry analysis.

**Results:**

The radiomic signature showed good performance for predicting CLN status in training and validation cohorts, with area under curve of 0.873 and 0.831 respectively. A radiogenomic map was created with nine significant correlations between radiomic features and gene modules, and two of them had higher correlation coefficient. Among these, MEmeganta representing the upregulation of telomere maintenance *via* telomerase and cell-cell adhesion was correlated with ‘Rectlike’ and ‘deviation ratio of tumor tissue and normal thyroid gland’ which reflect the margin and the internal echogenicity of the tumor, respectively. MEblue capturing cell-cell adhesion and glycolysis was associated with feature ‘minimum calcification area’ which measures the punctate calcification. The hub genes of the two modules were identified by protein-protein interaction network. Immunohistochemistry validated that LAMC1 and THBS1 were differently expressed in metastatic and non-metastatic tissues (p=0.003; p=0.002). And LAMC1 was associated with feature ‘Rectlike’ and ‘deviation ratio of tumor and normal thyroid gland’ (p<0.001; p<0.001); THBS1 was correlated with ‘minimum calcification area’ (p<0.001).

**Conclusions:**

The radiomic signature proposed here has the potential to noninvasively predict the CLN status in PTC patients. Merging imaging phenotypes with genomic data could allow noninvasive identification of the molecular properties of PTC tumors, which might support clinical decision making and personalized management.

## Introduction

Papillary thyroid carcinoma (PTC), the most common malignant thyroid cancer, accounts for approximately 90% of all thyroid malignancies (1). PTC is characterized by frequent metastasis to cervical lymph nodes (CLNs), which occurs in approximately 30-90% of PTC cases (2). CLNs metastasis (CLNM) increases the likelihood of local recurrence and is associated with decreased survival in the high-risk group (3, 4). The presence of LNM at diagnosis has a significant impact on the surgical approach (5, 6). Therefore, preoperative evaluation of the CLNM status is of paramount significance in designing an optimal therapeutic schedule and assessing the prognosis of PTC patients (7).

Fine-needle aspiration (FNA) is an alternative way to confirm LN involvement; however, FNA is an invasive procedure, and 15–30% of FNA samples cannot be categorized as benign or malignant (8). Ultrasound (US) examination of the CLNs before surgery is recommended by the American Thyroid Association for patients with known or suspected thyroid nodules. Unfortunately, preoperative US has a relatively low sensitivity for detecting CLNM (9, 10). In particular, some small metastases or metastases in the central compartment may not appear as abnormal findings on US images (11). The development of metastasis depends on the biological behavior of the primary tumor. Thus, accurate preoperative assessment of CLN status is still a great challenge in PTC.

Radiomics, the high-throughput extraction of extensive quantitative features to transform medical images into utilizable data that could likely be used as diagnostic, predictive or prognostic biomarkers and support the clinical decision-making, has drawn increased attention in cancer research recently (12, 13). A series of studies have suggested that radiomics-based approach could greatly improve the predictive performance of LNM in various types of cancer (14–17). Furthermore, previous studies have also integrated radiological imaging data with genomic data, and demonstrated the relationship between radiomic features and the corresponding molecular profile (18, 19). Such studies may facilitate the characterization of the underlying genetic mechanism that regulate the development of LNM in cancers (20). To date, few studies have applied US radiomics to assess CLNM in PTC patients, nor have they further investigated the association between the radiomic features of PTC and the underlying molecular genotype.

Therefore, in this study, we aimed to establish a radiomic signature to predict the CLN status of patients with PTC using preoperative thyroid US images; and investigate the associations between the radiomic features and the underlying molecular characteristics of the tumor.

## Materials and Methods

### Patients

**Figure 1:**
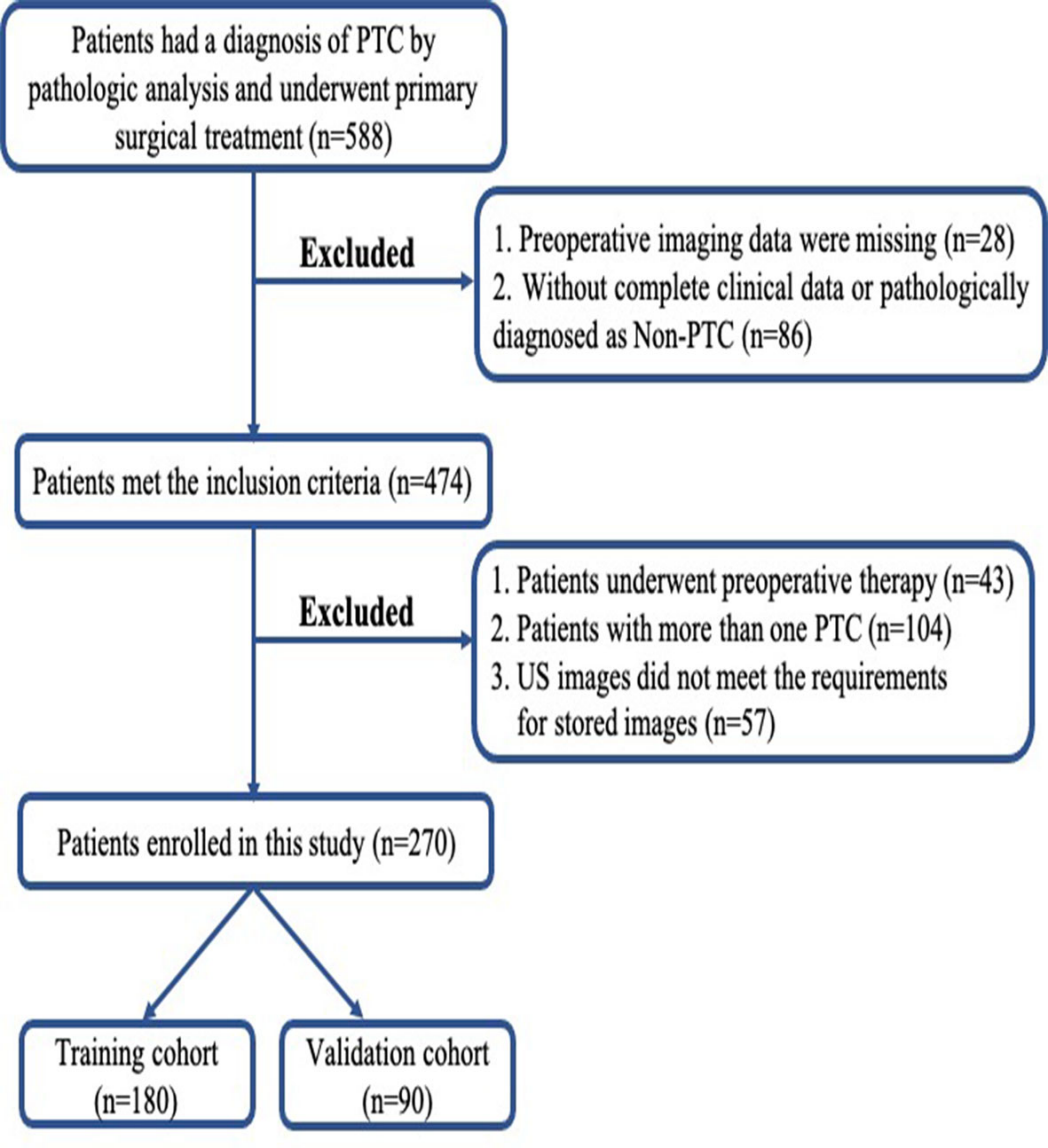
Flowchart of patient selection in this study. PTC, papillary thyroid carcinoma; US, ultrasound.

**Figure 2:**
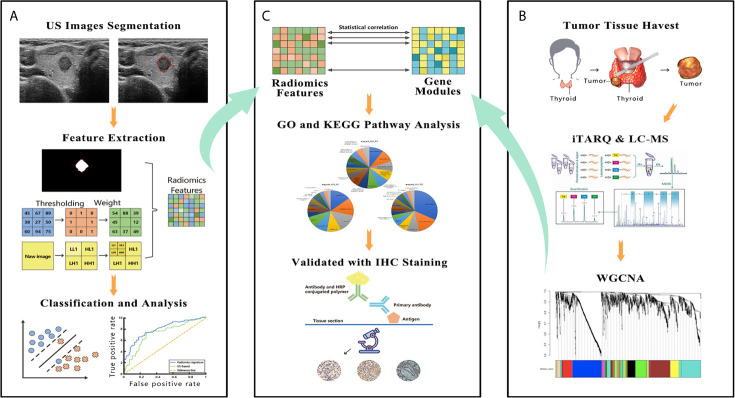
The figure shows the workflow and data processing of the radiogenomic pipeline. **(A)** US images were acquired before surgery, and these images were manually segmented. The radiomic features were extracted from segmented thyroid US images. A support vector machine was used to build the final radiomic signature. **(B)** A slice of tumor tissue was harvested during surgery and analyzed by iTRAQ and LC-MS. The whole bulk expression data were reduced to gene modules using WGCNA. **(C)** These modules were correlated with the radiomic features; then, the modules were further investigated by GO and KEGG pathway analyses. In the final step, IHC was performed to validate the results of the bioinformatic analysis. US, ultrasound; iTRAQ, isobaric tags for relative and absolute quantitation; LC-MS, liquid chromatograph-mass spectrometry; WGCNA, weighted gene co-expression network analysis; GO, Gene Ontology; KEGG, Kyoto Encyclopedia of Genes and Genomes; IHC, immunohistochemistry.

This prospective study was approved by the Ethics Committee of the hospital and complied with the *Declaration of Helsinki*. Written informed consent was provided by each patient. From January 2019 to December 2019, 588 consecutive patients with newly diagnosed thyroid tumors at our center took part in this study. All the patients underwent either lobectomy or total thyroidectomy. Dissection of the central CLNs was routinely performed, and if suspicious lateral LNs were indicated by FNA, preoperative CT or US, lateral LN dissection was performed. The inclusion criteria were as follows: i) All patients underwent neck ultrasound examination before surgery, and images were recorded and saved in DICOM format; ii) patients with complete clinical data and obtained the pathological diagnosis of PTC from the surgical specimen. The exclusion criteria included the following: i) preoperative therapy (radiofrequency or microwave ablation); ii) patients with multifocal lesions or bilateral disease; iii) poor imaging quality. After exclusion, 270 enrolled patients were divided into two data sets: 180 patients treated between January and August 2019 were assigned to the training set, whereas 90 patients treated between September and December 2019 were assigned to the validation set. The recruitment pathway for patients in the study was displayed in **Figure 1**. The pathological results of the LNs reviewed by senior pathologists with at least 6 years of experience were used as the gold standard for determining the LN status. **Figure 2** summarizes the workflow of the study design.

### US Evaluation of the CLNs

The status of the CLNs was assessed according to the ACR Thyroid Imaging, Reporting and Data System (TI-RADS) (21). In brief, a globular shape, the lack of a normal echogenic hilum, the presence of peripheral flow, heterogeneity with cystic components, and microcalcification were abnormal findings suggestive of CLNM. Based on these criteria, sonographic evaluation of the CLNs yielded a normal finding or suspected metastasis.

### US Imaging and Segmentation

All patients underwent US examination and image acquisition within one week before surgery. US images were acquired using ultrasonic equipment from Aixplorer (SuperSonic Imagine, Aix-en-Provence, France) with a 5-14 MHz linear transducer operated by radiologists with more than 6 years of experience. The US acquisition parameters were consistent among patients: gain, 53%; image depth, 3cm; focus parallel to the lesion. The spatial resolution of axial and lateral was 0.2 mm and 0.4 mm, respectively. The requirements for US Images were described elsewhere (22). Thyroid US images were recorded and saved in DICOM format, in which tumor images were manually segmented by other two clinicians with at least 4 years of experience in thyroid US. For each nodule, only the grey-scale image of the longitudinal section along the longest axis was subjected to segmentation. MATLAB R2015b software (MathWorks, Natick, USA) was used for manual segmentation. The image grayscale was normalized from 0 to 255 before performing radiomics feature extraction.

### Feature Extraction

All radiomic features recorded on the original B-mode US images comprised demographic information and tumor parenchyma-related features, which can reflect the size, shape, position, margin, echo pattern and calcification of the tumor, according to the ACR, ATA and American Association of Clinical Endocrinologists guidelines (10, 21, 23). The software “PTC cervical LNM prediction system” was used for feature extraction (22). The interclass correlation coefficient (ICC) was used to assess the inter- and intra-observer agreement of the feature extraction. An ICC > 0.80 was considered excellent and retained for subsequent analysis. Then, a 4-step selection method was employed to select the most effective features. The detailed procedures of the feature selection were performed as previously described (22, 24). All image and data processing were performed with MATLAB software. The details of the 50 features in the final feature set are listed in **Table S1**.

Redundant radiomic features were eliminated using a correlation matrix method in the radiomics-genomic correlation portion. For each individual feature, the mean absolute correlation based on pairwise correlation was calculated. If a pairwise correlation was beyond 0.8, features with the higher mean absolute correlation were removed. This process was conducted using the ‘findCorrelation’ function in the caret package in *R*.

### Classification

Many common classifiers are used in radiomics, and a support vector machine (SVM) is currently widely used because of its robustness and stability. In this study, we used an SVM for classification, and the specific mathematical model of the SVM was written as follows:

$$\underset{\omega ,b,\xi }{\min}\frac{1}{2}{\left||\omega |\right|}^{2}+C\sum_{i=1}^{N}\xi_{i}$$

$$s.t.y_{i}(\omega x_{i}+b)\geq 1-\zeta_{i},i=1,2,\ldots ,N,$$

$$\xi_{i}\geq 0,\;\;i=1,2,\ldots \;N,$$

where *ξ_i_* is the slack variable, *b* is the bias vector, *ω* is the weight vector, and *C* is the penalty parameter. In addition, xi ∈ X ∈ Rn, yi ∈ Y ∈ Rn, i=1, 2, …, N, where xi is the ith feature and yi is the label of xi.

A receiver operating characteristic (ROC) curve was created to assess the overall performance of the radiomic signature and the US-based method. Other criteria, such as the accuracy (ACC), sensitivity (SEN), specificity (SPEC) and area under the ROC (AUC), were calculated to evaluate the ability of the model to discriminate the LN status.

### Samples and Protein Extraction

Fifty-five tumor samples were obtained intraoperatively as the specimen was resected from the patients. A tumor slice from the the longitudinal section along the longest axis of the specimen was harvested (the slice thickness varied depending on the overall dimensions of the tumor). For larger nodules, we avoided any areas of overt central necrosis. Samples collected after surgery were frozen in liquid nitrogen until protein extraction. Each sample was homogenized in RIPA buffer and then subjected to ultrasonication. The resulting homogenates were held on ice for 30 min and centrifuged to remove the precipitates. The protein concentration of each sample was determined with a bicinchoninic acid (BCA) assay. Protein digestion was performed according to the filter-aided sample preparation (FASP) strategy described in a previous report (25). Briefly, 150 μg of protein was reduced, alkylated, and digested in a centrifuge tube. After the peptide solutions were digested at 37°C overnight, they were centrifuged, and the filtrates were collected. Finally, the digested peptides were dried by vacuum centrifugation and stored at -80°C until further use.

### iTRAQ Labeling

Seventy-five micrograms of peptides from each sample were used for iTRAQ labeling (AB SCIEX, Darmstadt, Germany). The labeling procedure was conducted in line with the manufacturer’s protocol with some modifications. After reconstitution in dissolution buffer, the digested peptides were incubated with a specific iTRAQ for 3 hours at room temperature. The labeled samples were homogenously mixed and dried by SpeedVac before they were redissolved in 60 μL of 5 mM ammonium formate. Next, 50 μL of the sample was prefractionated by high-pH reverse-phase liquid chromatography on an ACQUITY UPLC H-Class Bio system (Waters, Milford, MA, USA), and, finally, 10 consolidated fractions were obtained. The labeled peptides in each fraction were dried and redissolved in 30 μL of 2% acetonitrile/0.1% formic acid for LC-MS/MS analysis.

### Nano-LC-MS/MS

The labeled peptide mixtures were separated using an EASY-nLC 1000 system (Thermo Fisher Scientific, USA) and then trapped on a PepMap100 C18, 3 μm, 75 μm × 20 mm column (Thermo Fisher Scientific, USA). Then, the peptides were separated on a PepMap100, C18 2 μm 75 μm × 150 mm analytic column (NanoViper, Thermo Fisher Dionex, USA) with a 105 min mobile phase gradient from 5% to 35%. Mass spectra were recorded on a Q Exactive mass spectrometer equipped with a Nano-ESI source (Thermo Fisher Scientific, USA). Full-scan mass spectra were acquired in the 300-1600 m/z range at a resolution of 70,000, the top 20 precursors were selected for high-energy collision-induced dissociation (HCD) with a collision energy of 27%, and the productions were detected at a resolution of 17,500 on the data-dependent acquisition mode.

### iTRAQ Data Analysis

Protein sequences were searched using the MASCOT engine (version 2.3.2, Matrix Science, London, UK) embedded into Proteome Discoverer Software 1.4 (Thermo Electron, San Jose, USA). The following options were used for the search parameters: 1) database, SwissProt; 2) Taxonomy, *Homo sapiens*; 3) Enzyme, trypsin; 4) Fixed modifications, carbamidomethyl (C), iTRAQ 8-plex (N-term), iTRAQ 8-plex (K); 5) Variable modifications, oxidation (M) and iTRAQ 8-plex (Y); 6) Max missed cleavages, 2; 7) Peptide charge state, 2+, 3+, and 4+; 8) Peptide mass tolerance, 10 ppm; and 9) MS/MS tolerance, ± 0.02 Da. All reported data are based on 99% confidence for protein identification as determined by a false discovery rate (FDR) of ≤1%. The procedure was described in more detail by Plubell et al. (26) The data were analyzed with the support of Wayen Biotechnologies Co., Ltd. (Shanghai, China).

### Identification of Tumor Tissue Gene Modules

The large amount of gene expression data, complex potential combinations and multiple testing issues make traditional analytical methods infeasible. Weighted gene co-expression network analysis (WGCNA) is a well-established method that has become the standard in systems biology to identify modules—that is, clusters of genes with highly similar expression data. Compared with conventional clustering approaches, WGCNA is more robust and reliable because it considers both topological and correlative information. We employed the WGCNA package (version 1.64) in *R* software to identify gene modules. A detailed procedure for WGCNA was published in a previous study (27). Each gene module, also known as the module eigengene (ME), was summarized by its first principal component of the scaled (standardized) module expression profiles, and the minimum number of genes per module was set to 20.

### Clinical Relevance of Gene Modules

To investigate the correlation between gene modules and LNM, ROC curve analysis was used for tumor sample gene expression. Gene modules that had a relatively high diagnostic performance for LNM (area under ROC curve (AUC) >0.5) were selected for further analyses. MedCalc software was employed to analyze the ROC curves.

### Radiogenomic Correlations and Biological Function Annotations

The correlation matrix between the diagnostic gene modules and the selected radiomic features was built using Spearman rank correlations. Consequently, significant pairwise correlations between the radiomic features and the gene modules were obtained, and only the significant correlations were used in subsequent analyses.

To understand the biological functions of the genes listed in the gene modules, the Gene Ontology (GO) database was used to determine the biological properties of the identified genes (DAVID, http://david.abcc.ncifcrf.gov/). GO can be divided into three categories, namely, biological process, cellular component and molecular function. Pathway analysis was performed using the Kyoto Encyclopedia of Genes and Genomes (KEGG) database. For correcting the multiple hypothesis tests, the Benjamin-Hochberg method was used to control the FDR. The protein-protein interaction (PPI) network within the two modules was constructed with Cytoscape software using the online STRING database (version 10.5; http://string−db.org/). The top five most connected genes in the PPI network were defined as hub genes.

### Immunohistochemical Staining for LAMC1 and THBS1

In 40 cases, resected PTC tissue was fixed, embedded in paraffin, sectioned at 5 μm, and subjected to IHC. The sections were deparaffinized and quenched in a 3% hydrogen peroxide aqueous solution to block endogenous peroxidase activity. Then, antigen retrieval was performed using an antigen retrieval unmasking solution (Vector Laboratories, US). The expression levels of laminin subunit gamma 1 (LAMC1) and thrombospondin 1 (THBS1) were detected with anti- LAMC1 (HPA001908, Sigma-Aldrich, US) and THBS1 (ab1823, Abcam, US) antibodies, respectively. The sections were then counterstained with hematoxylin, dehydrated and mounted. Images of representative fields were visualized and photographed using a microscope (Leica, Germany) at ×10 and ×40 magnification.

### Evaluation of the Immunostained Sections

After immunohistochemical staining, the slides were reviewed and scored by an experienced pathologist. The intensity of LAMC1 and THBS1 expression was graded as follows: negative (-), weakly positive (+), moderately positive (++), and strongly positive (+++). Quantitative analysis of the captured images was performed with ImageJ software using the publicly available IHC toolbox plugin. The ratio of the positively stained area to the total area of each image was recorded as the IHC staining score.

### Statistical Analysis

Continuous variables are expressed as mean ± standard deviation (SD), Student’s t-test and Mann-Whitney U was used for comparisons between two groups. Categorical variables were compared by Mann-Whitney U or chi-squared test. The Pearson correlation coefficient *r* was used to assess the correlation between the value of radiomic features and the IHC staining score. To evaluate the diagnostic performance of radiomic and US in predicting CLNM, ROC analysis was performed, and the AUC was compared using Delong’s test. All statistical analyses were performed using SPSS software 23.0 for Mac (IBM Corporation, Armonk, NY), MedCalc software (version 19.0.4, Mariakerke, Belgium) or *R* (*R* Foundation for Statistical Computing, Vienna, Austria). To adjust for multiple comparisons, Benjamini–Hochberg method was used to control the false discovery rate (FDR). A two-tailed p-value<0.05 or FDR<0.1 was considered statistically significant.

## Results

### Patient Characteristics

The patients’ clinical characteristics in the training and validation sets are summarized in **Table S2**. No significant differences were found between the cohorts regarding the clinical characteristics. **Table 1** shows the associations between LNM positivity and the clinical parameters. Significant differences were found in terms of the patients’ age, tumor diameters, extrathyroidal extension (ETE), US assessment of CLN and the radiomic score.

**Table 1:** Clinical characteristics of PTC patients with or without LNM.

Characteristics	Training cohort			Validation cohort		
	Metastasis (n=81)	Non-Metastasis (n=99)	P value	Metastasis (n=43)	Non-Metastasis (n=47)	P value
Age			**0.001**			**0.033**
Mean ± SD	38.81 ± 11.42	43.90 ± 10.19		37.63 ± 11.72	42.64 ± 10.16	
Range	20-68	18-65		23-65	25-63	
Diameter			**0.002**			**0.002**
Mean ± SD	1.18 ± 0.61	0.90 ± 0.44		1.17 ± 0.58	0.85 ± 0.42	
Range	0.4-3.0	0.3-2.3		0.5-2.6	0.3-2.0	
Sex (%)			0.062			0.156
Male	30 (37.0)	24 (24.2)		17 (25.5)	12 (39.5)	
Female	51(63.0)	75 (75.8)		26 (74.5)	35 (60.5)	
Location (%)			0.694			0.815
Left	28 (37.8)	40 (40.4)		17 (44.7)	21 (39.5)	
Right	49 (57.2)	54 (54.5)		23 (46.8)	22 (53.5)	
Isthmus	4 (5.0)	5 (5.1)		3 (8.5)	4 (7.0)	
Hashimoto thyroiditis			0.988			0.507
Negative	50 (61.7)	61 (61.6)		24 (55.8)	28 (49.1)	
Positive	31 (38.3)	38 (38.4)		19 (44.2)	29 (50.9)	
ETE			**<0.001**			**<0.001**
Yes	58 (71.6)	9 (9.1)		30 (69.8)	7 (14.9)	
No	23 (28.4)	90 (90.9)		13 (30.2)	40 (81.5)	
US assessment of CLN			**0.012**			**0.011**
Positive	32 (39.5)	22 (22.2)		20 (46.5)	10 (21.3)	
Negative	49 (60.5)	77 (77.8)		23 (53.5)	37 (78.7)	
Radiomic score			**<0.001**			**<0.001**
Median	0.697	-0.790		0.627	-0.948	
Interquartile range	(0.201, 1.055)	(-1.09, -0.190)		(0.108, 1.053)	(-1.294, -0.232)	

PTC, papillary thyroid carcinoma; LNM, lymph node metastases; SD, standard deviation; US, ultrasound; CLN, cervical lymph nodes; ETE, extrathyroidal extension; Significant differences are highlighted in boldface.

### Prediction of CLN Status Based on Radiomic Evaluation

**Figure 3:**
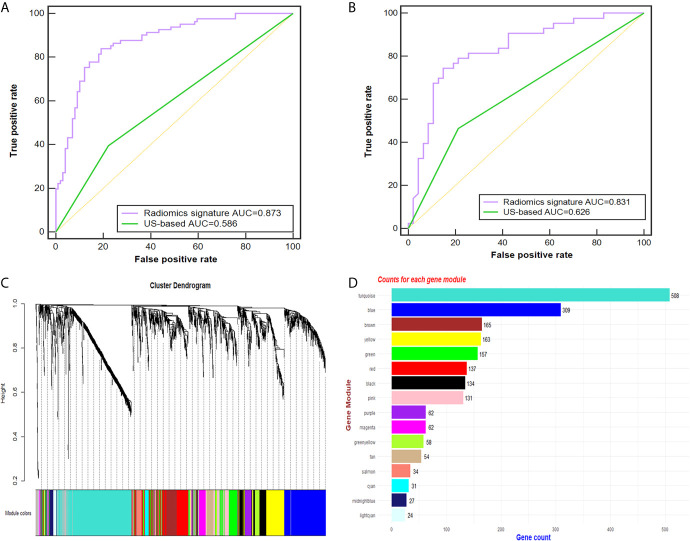
Radiomic signature performance and WGCNA analysis. **(A, B)** The ROC curves of the radiomic signature and the US-based method in the training **(A)** and validation cohorts **(B)**. Cluster dendrogram of 2137 proteins for 49 PTC patients created *via* the WGCNA method. **(C)** Gene dendrogram after clustering; each color represents one gene module. **(D)** Counts for each gene module. AUC, area under the receiver operating characteristic curve; US, ultrasound; PTC, papillary thyroid carcinoma; ROC, receiver-operating characteristic; WGCNA, weighted gene co-expression network analysis.

Of the 614 features extracted from the selected ROIs of the training cohort, 35 features with an ICC <0.8 were excluded. After feature selection, finally, the radiomic signature was built with 50 effective features *via* the support vector machine and was applied to predict the CLN status in both cohorts. The diagnostic performance of the radiomic signature was assessed by ROC curve analysis (**Figure 3**). The radiomic signature yielded an accuracy (ACC), sensitivity (SEN), specificity (SPEC), and AUC of 82.2%, 83.6%, 80.8% and 0.873, respectively, in the training cohort; and 80.0%, 74.4%, 85.1% and 0.831, respectively, in the validation cohort. In contrast, assessment of the CLN status based solely on grey-scale US yielded an ACC, SEN, SPEC and AUC of 60.6%, 39.5%, 77.8% and 0.586, respectively, in the training cohort; and 63.3%, 46.5%, 78.7% and 0.626, respectively, in the validation cohort. The AUC of the radiomic signature was significantly higher than that of the US-based method (p<0.001).

### Genomic Module Clustering and ROC Analysis

Twenty-six tumor tissues with CLNM and twenty-nine tissues without CLNM collected from PTC patients were used for iTRAQ assessment. Due to protein degradation or uncertainty of the pathological report, 6 samples were excluded from further analysis with subsequent bioinformatic analysis focused on the remaining 49 tumor samples. After the tissues were processed for LC-MS, a total of 2137 proteins were identified after rejecting proteins with unreliable expression. Sixteen gene modules were generated by WGCNA. **Figures 3C, D** shows the cluster dendrogram and gene counts for each module, respectively. ROC analysis was employed to evaluate the discriminatory power of each module for predicting CLNM. The AUC values for each module were between 0.354 and 0.807. Among the 16 modules, eight had good discriminatory performance (AUC greater than 0.5) and were selected for further study (**Table 2**).

**Table 2:** AUC for the gene modules in assessing cervical lymph node metastasis.

Variable	AUC	95% CI
MEmidnightblue	0.807 ± 0.079	0.636-0.922
MElightcyan	0.804 ± 0.078	0.632-0.919
MEpink	0.709 ± 0.093	0.528-0.851
MEmagenta	0.719 ± 0.093	0.539-0.859
MEsalmon	0.688 ± 0.010	0.506-0.835
MEblue	0.691 ± 0.095	0.510-0.838
MEtan	0.614 ± 0.106	0.432-0.775
MEcyan	0.589 ± 0.109	0.408-0.755
MEpurple	0.470 ± 0.101	0.272-0.668
MEblack	0.467 ± 0.103	0.264-0.669
MEgreenyellow	0.435 ± 0.100	0.240-0.630
MEturquoise	0.425 ± 0.105	0.219-0.630
MEred	0.421 ± 0.106	0.214-0.629
MEgreen	0.418 ± 0.102	0.217-0.618
MEbrown	0.397 ± 0.099	0.203-0.590
MEyellow	0.354 ± 0.096	0.165-0.543

AUC, area under the curve; CI, confidence interval.

### Associations Between Radiomic Features and Gene Modules

**Figure 4:**
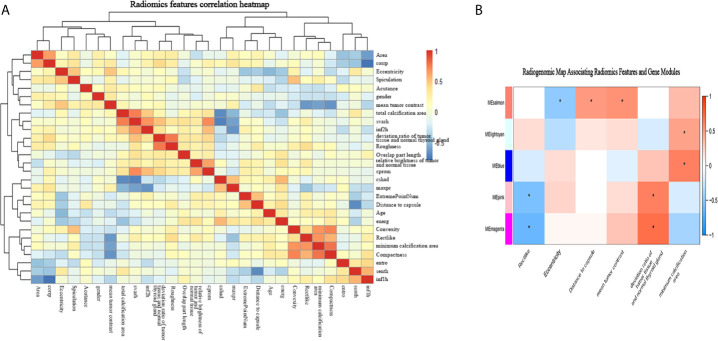
The radiogenomic correlation map. **(A)** The correlation matrix of the 28 radiomic features. **(B)** The radiogenomic correlation map between the selected radiomic features and the gene modules created using the Spearman rank correlation method; significant correlations (p<0.05) are identified with asterisks. The corresponding coefficients are displayed in a heatmap, where red and blue indicate positive and negative correlations, respectively. *, a statistically significant association.

Among the 50 selected radiomic features, 28 remained after redundancy removal. The correlation matrix of the remaining radiomic features is shown in **Figure 4A**. A radiogenomic map was constructed by correlating the selected radiomic features and the gene modules. Nine pairwise correlations were statistically significant in the radiogenomic correlation map, as identified by an asterisk (**Figure 4B**). For example, we found MEmeganta which capturing the upregulation of telomere maintenance *via* telomerase and cell-cell adhesion was associated with radiomic features ‘deviation ratio of tumor tissue and normal thyroid gland’ and ‘Rectlike’. When this module was active, the lesion tended to be more hypoechoic or even markedly hypoechoic with an ill-defined margin. Whereas, when this module was inactive, the lesion tended to be isoechoic and have a relatively smooth margin. In addition, the module MEblue enriched with genes associated with cell-cell adhesion and glycolysis had significant correlation with ‘minimum calcification area’, which indicated that genes active in this module was positively correlated with punctate calcification. Overall, our radiogenomic analysis captured multiple associations between radiomic features and molecular characteristics in PTC.

Since module MEmagenta and MEblue had a higher correlation coefficient with the radiomic features according to the radiogenomic correlation map, we focused more deeply on these two modules.

### GO and KEGG Pathway Enrichment Analyses of the Gene Modules

**Figure 5:**
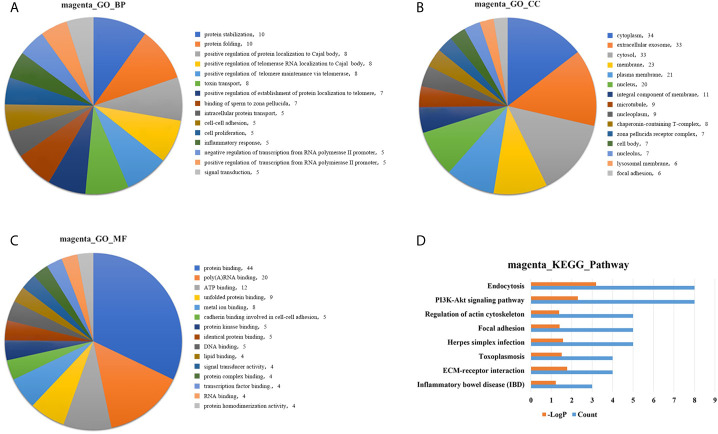
Top significantly enriched GO terms of the MEmagenta module, including **(A)** biological process; **(B)** cellular component; and **(C)** molecular function. Significantly enriched pathways identified by KEGG pathway analysis. **(D)** Pathways enriched in the MEmagenta module. The detailed statistical data were shown in the **Supplementary Data Sheet**. GO, Gene Ontology; KEGG, Kyoto Encyclopedia of Genes and Genomes.

**Figure 6:**
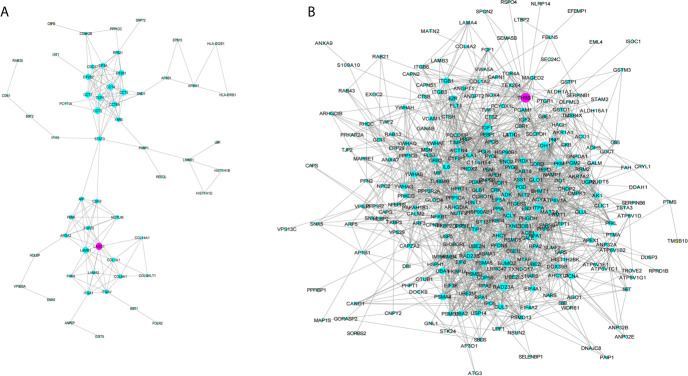
Protein-protein interaction (PPI) network of the genes listed in the modules MEmegenta **(A)** and MEblue **(B)**. Proteins selected for further immunohistochemistry validation are shown in purple.

GO analysis was applied to determine the biological process, cellular component, and molecular functions of the MEmagenta (**Figures 5A–C**) and MEblue (**Figure S1A–C**) modules, and KEGG analysis was performed to investigate the involved signaling pathways of the two modules. The most significantly enriched pathways are shown in **Figures 5D** and **S1D**, and the protein-protein interaction (PPI) network of the two modules is shown in **Figure 6**. In the MEmagenta module, the hub genes were *CCT3, TCP1, CCT4, LAMB1* and *LAMC1*. In the MEblue module, the hub genes were *RAD23A, PGD, ITPA, IDH1 and THBS1*. **Table 3** lists the annotations of the three other gene modules as GO terms and KEGG pathway outputs.

**Table 3:** Annotations of significant gene modules in GO terms (biological process, cellular component and molecular function) and KEGG pathway.

Gene module	GO term (biological Process) (adjust p<0.05)	GO term (Cellular Component) (adjust p<0.05)	Go term (Molecular Function) (adjust p<0.05)	KEGG pathway (adjust p<0.05)	Abbreviation
MElightcyan	Regulation of complement activation	Membrane attack complex	Phospholipid binding	Complement and coagulation cascades	Complement activation Protein binding
	Complement activation (alternative pathway)	Extracellular region	Structural constituent of cytoskeleton	Proteoglycans in cancer	
	Complement activation (classical pathway)	Spectrin-associated cytoskeleton	Receptor binding	PPAR signaling pathway	
MEpink	DNA topological change	Nucleoplasm	RNA binding	Ribosome biogenesis in eukaryotes	Cell division
	Chromatin remodeling	Membrane	Protein binding	Complement and coagulation cascades	
	mRNA splicing	Nucleolus	Chromatin binding	RNA transportation	
MEsalmon	Cell-cell adhesion	Extracellular matrix	Heparin binding	Proteoglycans in cancer	Cell adhesion and migration
	Extracellular matrix organization	Extracellular space	Collagen binding	ECM-receptor interaction	
	Endothelial cell migration	Extracellular region	Extracellular matrix binding	Focal adhesion	

### Validation of the Bioinformatic Analysis Results by IHC

**Figure 7:**
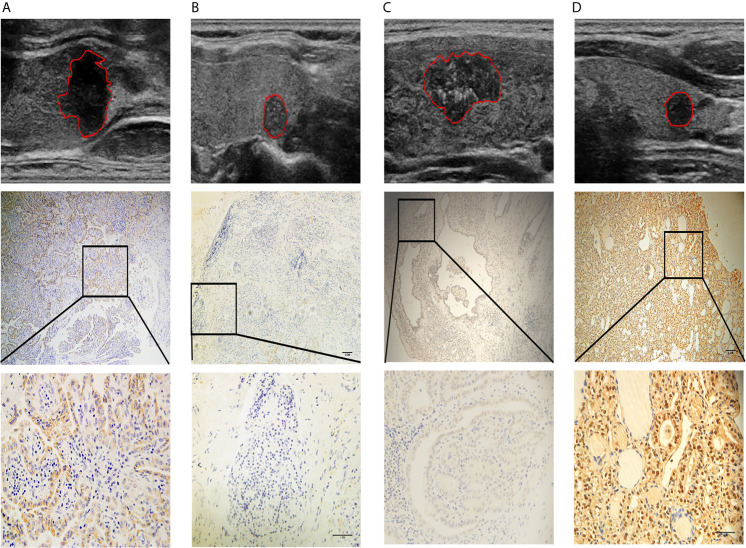
Four representative cases are shown: **(A)** a lesion with a smooth margin, relatively hyperechoic signal, and low LAMC1 expression level, which was confirmed by immunohistochemical staining; **(B)** a lesion with a lobulated/irregular margin, hypoechoic signal and relatively high LAMC1 expression level; **(C)** a lesion with little microcalcification and a relatively high THBS1 expression level, which was confirmed by immunohistochemical staining; and **(D)** a lesion with much more microcalcification and relatively low THBS1 expression. In each case, the tumor images are displayed at 10X and 40X magnification.

Next, we performed IHC on 40 of the samples tested by iTRAQ and LC/MS to further validate the putative connections between the radiomic features and biological information. *LAMC1* and *THBS1* were identified as hub genes for MEmagenta and MEblue, respectively. These two modules were both inclusive of the PI3K/AKT signaling pathway, and *LAMC1* and *THBS1* are both involved in the PI3K/AKT pathway. Therefore, we selected these two representative genes from each module for further validation (**Figure 7**).

The results of immunohistochemical staining for LAMC1 and THBS1 are shown in **Table 4**. Significant differences in LAMC1 and THBS1 staining intensity were found between the PTC tissues with and those without CLNM (U=93.0, p=0.003; U=90.0, p=0.002).

**Table 4:** LAMC1 and THBS1 expression in PTC with or without cervical lymph node metastasis.

Gene	Pathology	-	+	++	+++	Total	U	P value
LAMC1	Non-metastatic PTC	7	8	3	2	20	93	0.003
	Metastatic PTC	1	5	7	7	20		
THBS1	Non-metastatic PTC	1	4	8	7	20	90	0.002
	Metastatic PTC	6	9	3	2	20		

PTC, papillary thyroid carcinoma.

The values of the radiomic features ‘Rectlike’ and ‘deviation ratio of tumor and normal thyroid gland’ were significantly correlated with the expression of LAMC1 (r=-0.658, p<0.001; r=0.715, p<0.001). In addition, the value of the feature ‘minimum calcification area’ was highly correlated with the expression of THBS1 (r=-0.756, p<0.001).

## Discussion

In this study, we developed a radiomic signature to predict the CLNM of patients with PTC using preoperative thyroid US images, and integrated the radiomic features with the gene expression profile to identify potential radiogenomic biomarkers for PTC. We successfully identified 9 significant pairwise associations between radiomic features and gene modules annotated by functional gene enrichment analysis. These associations demonstrated the feasibility of the noninvasive molecular characterization of PTC using radiogenomic methods, which might provide complementary information for the noninvasive classification and management of PTC.

High-resolution US is regarded as the first choice for the preoperative assessment of the CLN status; however, the efficiency of US in detecting nonpalpable CLNM is unacceptable. In particular, metastatic nodes in the central compartment are not easily detected by US examination because they are obscured by the complex structures in the central neck. In our study, diagnosing CLNM based solely on conventional US yielded an AUC of only 0.586 and 0.626 in both cohorts, with relatively low ACC and SEN. Radiomics sometimes applies machine learning methods and has attracted the research interest of many scholars who seek to explore the association between diagnostic information and imaging features (28). The AUC of our radiomic signature was improved to 0.873 and 0.831, which indicated that compared with the model relying solely on the US examination of LNs, the radiomic model had better performance in predicting CLNM, which is consistent with our previous work (24).

The feature ‘Rectlike’ measures the smoothness of the tumor margin, and a higher ‘Rectlike’ value indicates a smoother margin. An ill-defined margin was reported to be significantly associated with CLNM (29); therefore, patients with a higher ‘Rectlike’ value tended to have a lower risk of CLNM. ‘Rectlike’ was negatively correlated with the modules MEpink and MEmagenta. Upon conducting GO and KEGG pathway analysis, MEpink was enriched in cell division, while MEmagenta represented the upregulation of telomere maintenance *via* telomerase and cell-cell adhesion. Cancer progression is achieved by uncontrolled cell division, invasion, and, eventually, metastasis (30). Many anticancer agents have been designed to regulate cell division to curb cancer progression (31). Cell division causes the telomere to shorten gradually; hence, a key event in the acquisition of cellular immortality is upregulation of the telomere maintenance mechanism (32). Telomerase is an enzyme responsible for telomere maintenance. An important mechanism of telomerase activation includes mutations in the promoter region of the telomere reverse transcriptase (*TERT*) gene (33). Clinically, mutations in the *TERT* promoter are frequently examined from FNA samples (34). PTC tumors harboring TERT mutations showed more frequent regional LNM spread than did tumors with a wild-type TERT promoter (35), and TERT mutations are markers for metastatic behavior (36).

The shape feature ‘Eccentricity’ measures the ratio of the longitudinal axis to the horizontal axis of the tumor. Tumors with a higher ‘Eccentricity’ value tend to be taller than wide in shape. Previous studies have demonstrated that a taller-than-wide shape on US is an independent predictor for the absence of CLNM (37, 38). ‘Eccentricity’ was negatively correlated with the MEsalmon, which was related to cell-cell adhesion and extracellular matrix (ECM) in the GO and KEGG enrichment analyses. Cell-cell adhesion is essential for cell-cell cooperation, multicellular polarity, tissue homeostasis, and collective cell movement (39). Cancer cell migration, the basis for metastatic dissemination, is a plastic and adaptive process integrating cell-cell adhesion, cytoskeletal dynamics, and ECM remodeling. In single-cell migration, the loss of adhesion between cells triggers a dynamic change in the actin cytoskeleton, which endows the cells with motility and alters cell polarity to form spindle-shaped cells. These newly formed mesenchymal-like cells invade the basal ECM and migrate to the underlying tissues (40). Degradation and remodeling of the ECM, including the basement membrane, by tumor-secreted proteolytic enzymes are also crucial steps in the process of cancer cell intra- and extravasation and colonization at distant sites (41).

The position feature ‘Distance to capsule’ indicates the distance between the nodule and the nearest thyroid capsule. Evidence has shown that the tumor being in close proximity to or within the capsule was significantly more indicative of CLNM in PTC (29, 42). Closer proximity to the thyroid capsule may offer more chances for tumors to encounter lymphatic vessels, therefore increasing the likelihood of metastasis within the lymphatic system (38). ‘Distance to capsule’ was also correlated with MEsalmon.

Both ‘mean tumor contrast’ and ‘deviation ratio of tumor and normal thyroid gland’ are features that reflect the internal echogenicity of PTC. Tumors with higher ‘mean tumor contrast’ and ‘deviation ratio of tumor and normal thyroid gland’ values tend to be more hypoechoic or even markedly hypoechoic. Lee et al (43). demonstrated that hypoechoic and markedly hypoechoic tumors on US were independent risk factors for CLNM, and these malignant US appearances suggested a more invasive biological behavior, including CLNM (44). ‘Mean tumor contrast’ was correlated with the MEsalmon as well, and ‘deviation ratio of tumor and normal thyroid gland’ was correlated with the MEpink and MEmagenta.

Minimum calcification area’ is a feature that measures the extent of microcalcification of PTC. The higher the value, the more microcalcifications there are within the tumor. Microcalcification has been recognized as an independent predictive factor for CLNM (37). ‘Minimum calcification area’ was positively correlated with MElightcyan and MEblue. MElightcyan was related to complement activation, while MEblue represented cell-cell adhesion and glycolysis. Accumulating evidence has shown that complement activation in the tumor microenvironment promotes tumor growth, suppresses antitumor immunity, and increases metastasis (45). Enhanced glycolysis has been considered to be the dominant metabolic alteration in malignant tumors (46) and the primary source of ATP for tumor survival upon detachment and during metastasis (47).

Alterations in the expression levels of the genes of interest were confirmed by IHC, indicating that these molecules play an important role in the process of PTC metastasis. LAMC1 is reportedly involved in the progression of various malignant tumors. A study showed that the overexpression of LAMC1 in endometrial carcinoma was related to aggressive histological types and LN metastasis; and LAMC1 knockdown suppressed cell motile and invasive properties in endometrial cancer cells (48). Furthermore, treatment with the specific LAMC1 peptide enhanced pulmonary metastasis of B16 melanoma cells and induced the production of matrix metalloproteinase-9 from B16 cells (49). Consistent with previous studies, we found that LAMC1 was expressed at a relatively higher level in samples from patients with metastasis.

THBS1 is a secreted glycoprotein involved in tumor progression *via* the regulation of ECM remodeling and angiogenesis. The role of THBS1 as an antiangiogenic factor is well documented; however, its effect on tumor progression and metastasis remains controversial (50). On the one hand, Giuseppe and colleagues found a significant reduction in THBS1 expression associated with patients presenting thyroid carcinoma metastasis (51). *In vitro*, THBS1 inhibited the migration of clear cell renal carcinoma cells (52). On the other hand, THBS1 has been reported to promote human follicular thyroid carcinoma cell invasion through the upregulation of urokinase-dependent activity (53) and metastasis to the lungs in a transgenic mouse model of breast cancer (54). Our present data indicate that *THBS1* may function as a suppressor gene in PTC.

Radiomics could provide a more accurate and robust method to predict CLN involvement in PTC patients because the proteomic pattern is expressed in terms of image-based features. A previous study evaluated the association between the gene expression signature and CLNM in PTC (55); however, this approach is limited by the requirement of invasive procedure and its high cost. The imaging features in radiomics studies generally lack biological interpretations, which might hinder their clinical application. Thus, linking imaging characteristics with molecular signatures is a growing trend that provides additional value to conventional imaging with relevant molecular biological information. In this study, we identified radiomic features that were associated with gene modules, annotated the molecular and physiological effects of the relevant gene modules, and validated the associations and the results of the bioinformatic analysis, all of which lend convincing support to the proposed method.

Several limitations were encountered in our study. First, the application of multimodal imaging is common in radiomics, the use of elastography and contrast-enhanced US as other modalities may provide a more robust and discriminative signature. Second, to minimize the influence of variation in imaging acquisition, the US images were all acquired from single center using the same equipment. Third, the radiomic features used in this study were not Imaging Biomarker Standardization Initiative (ISBI) compliant. In our following studies, we are going to use the ISBI compliant features to improve the reproducibility. This may affect generalization of our results. A multi-center study using all kinds of US equipment will be needed in the future (56). Finally, we focused on molecular analysis at the protein level. In the future, by incorporating other types of ‘-omic’ data, we could provide a more complete picture of the molecular characteristics of tumors.

## Conclusion

The radiomic signature proposed here has the potential to noninvasively predict the CLN status in PTC patients. Merging imaging phenotypes with genomic data could allow noninvasive identification of the molecular properties of PTC tumors, which might further facilitate the application of radiomics in clinical practice for cancer patients.

## Data Availability Statement

The original contributions presented in the study are included in the article/**Supplementary Material**. Further inquiries can be directed to the corresponding authors.

## Ethics Statement

This prospective study was approved by the Ethics Committee of the hospital and complied with the Declaration of Helsinki. The patients/participants provided their written informed consent to participate in this study.

## Author Contributions

YT and PS performed the experiments, interpreted the data, and wrote the paper. JJY evaluated all the pathology slides. YH collected the clinical data and the ultrasound images. YG, JHY, and YYW contributed to feature extraction and model construction. YLW, YW, and QJ revised the whole article. SZ and CC conceived the idea and provided the funding support. HZ did the bioinformatic analysis. All authors contributed to the article and approved the submitted version.

## Funding

This study was supported by the National Natural Science Foundation of China (81401422, 82071945) and the Shanghai Science and Technology Foundation of China (17411963300).

## Conflict of Interest

The authors declare that the research was conducted in the absence of any commercial or financial relationships that could be construed as a potential conflict of interest.
