# Supplementary material for: Radiogenomic Analysis of Papillary Thyroid Carcinoma for Prediction of Cervical Lymph Node Metastasis: A Preliminary Study
Source: Front Oncol. 2021 Jun 29;11:682998. doi: 10.3389/fonc.2021.682998 (PMC8276635; doi:10.3389/fonc.2021.682998)
Supplement: Supplementary file 3 [file DataSheet_3.docx]

Supplementary Material

# Supplementary Tables

**Table S1**. Radiomics features of thyroid nodules

|  |  | Lymph Nodes Metastasis | | | | |
| --- | --- | --- | --- | --- | --- | --- |
|  |  | No | | Yes | |  |
| Feature Type | Feature Name | Median  (interquartile range) | | Median  (interquartile range) | | P value* |
| Demographic data | Sex |  |  |  |  |  |
|  | Age^#^ | 43.90 ± 10.19 | | 38.81 ± 11.42 |  | 0.005 |
| Size | Area | 0.24 (0.12-0.36)  0.50 (0.36-0.64) | | 0.42 (0.30-0.54)  0.56 (0.42-0.70) | | 0.003 |
|  | The ratio of the tumor area to that of its convex hull |  |  |  |  | 0.039 |
| Shape | Eccentricity | 0.97 (0.92-1.00)  0.50 (0.43-0.56)  0.10 (0.07-0.13)  0.92 (0.90-0.95) | | 0.91 (0.88-0.95)  0.62 (0.55-0.69)  0.11 (0.09-0.14)  0.74 (0.62-0.85) | | <0.001 |
|  | Convexity |  |  |  |  | 0.029 |
|  | Roundness |  |  |  |  | 0.007 |
|  | Compactness |  |  |  |  | <0.001 |
| Margin | Spiculation | 11.28 (5.46-16.65)  7.40 (6.24-8.51)  0.74 (0.62-0.85)  3.15 (2.00-3.99)  0.02 (0.01-0.04) | | 11.80 (5.64-16.88)  7.90 (6.37-8.64)  0.63 (0.52-0.76)  3.30 (2.03- 4.12)  0.04 (0.03-0.06) | | 0.660 |
|  | ExtremePointNum |  |  |  |  | 0.524 |
|  | Rectlike |  |  |  |  | <0.001 |
|  | Acutance |  |  |  |  | 0.320 |
|  | Roughness |  |  |  |  | <0.001 |
| Orientation | Elliptical-normalized eccentricity | 0.20 (0.13-0.27) | | 0.10 (0.04-0.15) | | <0.001 |
|  | Elliptical-normalized angle | 0.41 (0.28-0.56) | | 0.77 (0.60-0.92) | | <0.001 |
|  | Length-to-width ratio | 6.74 (4.35-8.89) | | 3.87 (2.01-5.96) | | <0.001 |
| Position | Overlap part length | 0.15 (0.12-0.17)  2.60 (2.11-3.15)  0.72 (0.57-0.87) | | 0.14 (0.12-0.19)  2.48 (2.03-3.00)  0.64 (0.49-0.79) | | 0.172 |
|  | Overlap part area |  |  |  |  | 0.046 |
|  | Distance to capsule |  |  |  |  | 0.005 |
| Echo pattern | mean tumor contrast | 0.45 (0.24-0.66) | | 0.48 (0.25-0.65) | | 0.056 |
|  | mean tumor covariance | 0.02 (0.01-0.03) | | 0.02 (0.01-0.004) | | 0.3111 |
|  | mean tumor non-similarity | 0.99 (0.45-1.53) | | 1.01 (0.47-1.50) | | 0.835 |
|  | deviation ratio of tumor tissue and normal thyroid gland | 0.51 (0.30-0.73) | | 0.67 (0.40-1.05) | | <0.001 |
|  | relative brightness of tumor and normal tissue | 0.07 (0.05-0.09) | | 0.06 (0.05-1.00) | | 0.190 |
|  | relative brightness of tumor and normal muscle | 0.08 (0.04-0.13) | | 0.09 (0.05-0.14) | | 0.096 |
|  | Autoc | 11.00 (6.65-15.98) | | 9.32 (5.85-12.87) | | 0.008 |
|  | Contr | 0.43 (0.20-0.60) | | 0.31 (0.13-0.50) | | <0.001 |
|  | Corm | 0.89 (0.79-1.01) | | 0.93 (0.82-1.03) | | 0.018 |
|  | Corrp | 0.90 (0.81-1.05) | | 0.93 (0.80-1.10) | | 0.018 |
|  | Cprom | 257.51 (142.28-360.11) | | 249.46 (144.36-369.41) | | 0.485 |
|  | Cshad | 0.84 (0.61-1.07) | | 0.78 (0.58-1.02) | | 0.066 |
|  | Dissi | 0.22 (0.13-0.30) | | 0.18 (0.11-0.28) | | <0.001 |
|  | Energy | 0.16 (0.09-0.23) | | 0.22 (0.15-0.29) | | <0.001 |
|  | Entro | 2.15 (1.83-2.55)  0.89 (0.81-0.98)  0.95 (0.84-1.07)  0.28 (0.17-0.40)  11.45 (7.76-16.21)  23.24 (10.08-26.37)  25.11 (14.12-36.39)  1.93 (1.63-2.40)  0.41 (0.18-0.64)  0.52 (0.41-0.63)  0.93 (0.82-1.04)  -0.65 (-0.73, -0.57) | | 2.02 (1.73-2.30)  0.96 (0.89-1.07)  0.87 (0.82-1.04)  0.38 (0.28-0.49)  9.68 (6.06-13.50)  21.12 (9.56-25.32)  22.41 (12.96-32.01)  1.85 (1.57-2.05)  0.31 (0.15-0.48)  0.47 (0.38-0.56)  0.94 (0.81-1.05)  -0.71 (-0.77, -0.62) | | 0.005 |
|  | Homom |  |  |  |  | 0.044 |
|  | Homop |  |  |  |  | <0.001 |
|  | Maxpr |  |  |  |  | <0.001 |
|  | Sosvh |  |  |  |  | 0.007 |
|  | Savgh |  |  |  |  | 0.005 |
|  | Svarh |  |  |  |  | 0.017 |
|  | Senth |  |  |  |  | 0.014 |
|  | Dvarh |  |  |  |  | <0.001 |
|  | Denth |  |  |  |  | <0.001 |
|  | inf1h |  |  |  |  | 0.495 |
|  | inf2h |  |  |  |  | <0.001 |
| Calcification | total calcification area | 0.98 (0.87-1.09)  0.97 (0.95-0.99)  12.42 (4.95-20.53)  25.27 (5.18-44.67)  328.50 (257.83-414.14) | | 1.00 (0.88-0.10)  0.96 (0.94-1.00)  16.32 (6.71-24.33)  26.11 (5.65-43.21)  331.46 (259.66-418.55) | | 0.354 |
|  | maximum calcification area |  |  |  |  | 0.262 |
|  | minimum calcification area |  |  |  |  | <0.001 |
|  | total calcification circumference |  |  |  |  | 0.947 |
|  | the number of calcification point |  |  |  |  | 0.162 |

SD indicates standard deviation; ^#^Age is presented as the mean ± SD, and was compared by the Student t test; *p values were FDR adjusted.

**Table S2.** Baseline characteristics of the PTC patients

| Characteristics | Training cohort  (n=180) | Validation cohort  (n=90) | P value |
| --- | --- | --- | --- |
| Age (years) |  |  | 0.340 |
| Mean ± SD | 41.61 ± 11.02 | 40.24 ± 11.16 |  |
| Range | 18-68 | 23-65 |  |
| Diameter (cm) |  |  | 0.723 |
| Mean ± SD | 1.03 ± 0.54 | 1.00 ± 0.52 |  |
| Range | 0.3-3.0 | 0.3-2.6 |  |
| Sex |  |  | 0.709 |
| Male | 54 (34.9) | 29 (32.6) |  |
| Female | 126(65.1) | 61 (67.4) |  |
| Location |  |  | 0.437 |
| Left | 68 (37.8) | 38 (42.2) |  |
| Right | 103 (57.2) | 45 (50.0) |  |
| Isthmus | 9 (5.0) | 7 (7.8) |  |
| Hashimoto thyroiditis |  |  | 0.538 |
| Negative | 111 (61.7) | 52 (57.8) |  |
| Positive | 69 (38.3) | 38 (42.2) |  |
| ETE |  |  | 0.536 |
| Yes | 67 (37.2) | 37 (41.1) |  |
| No | 113 (62.8) | 53 (28.9) |  |
| US assessment of CLN |  |  | 0.577 |
| Positive | 54 (30.0) | 30 (33.3) |  |
| Negative | 126 (70.0) | 60 (66.7) |  |
| Radiomic score |  |  | 0.696 |
| Median | -0.111 | -0.093 |  |
| Interquartile range | (-0.896, 0.776) | (-1.021, 0.791) |  |

PTC indicates papillary thyroid carcinoma; SD indicates standard deviation; US indicates ultrasound; ETE indicates extrathyroidal extension; CLN indicates cervical lymph nodes.

# Supplementary Figures

**
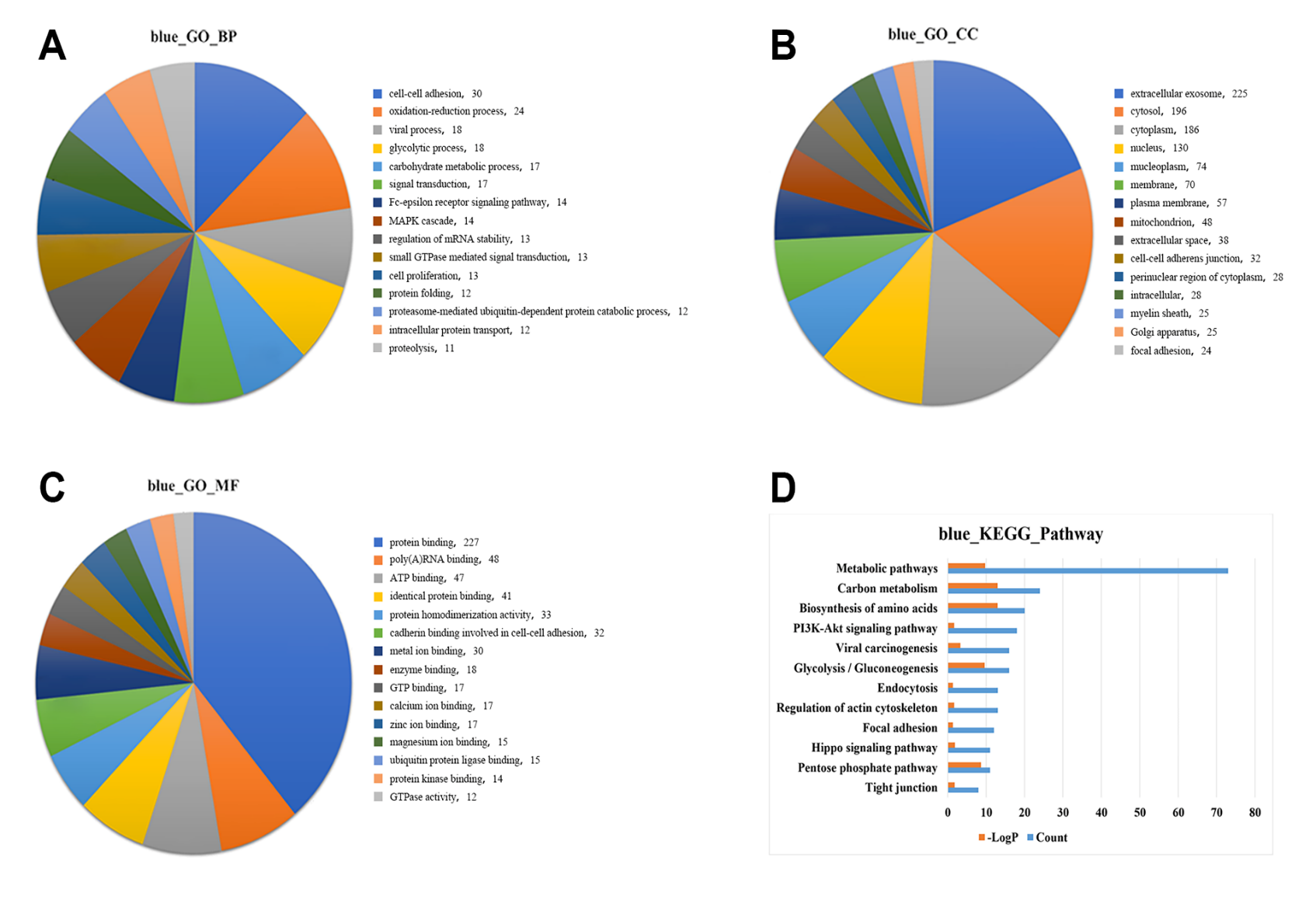
**

**Figure S1.** Top significantly enriched GO terms of the MEblue module, including A) biological process; B) cellular component; and C) molecular function. Significantly enriched pathways identified by KEGG pathway analysis. D) Pathways enriched in the MEblue module. The detailed statistical data were shown in the supplementary data sheet. GO indicates Gene Ontology; KEGG indicates Kyoto Encyclopedia of Genes and Genomes.


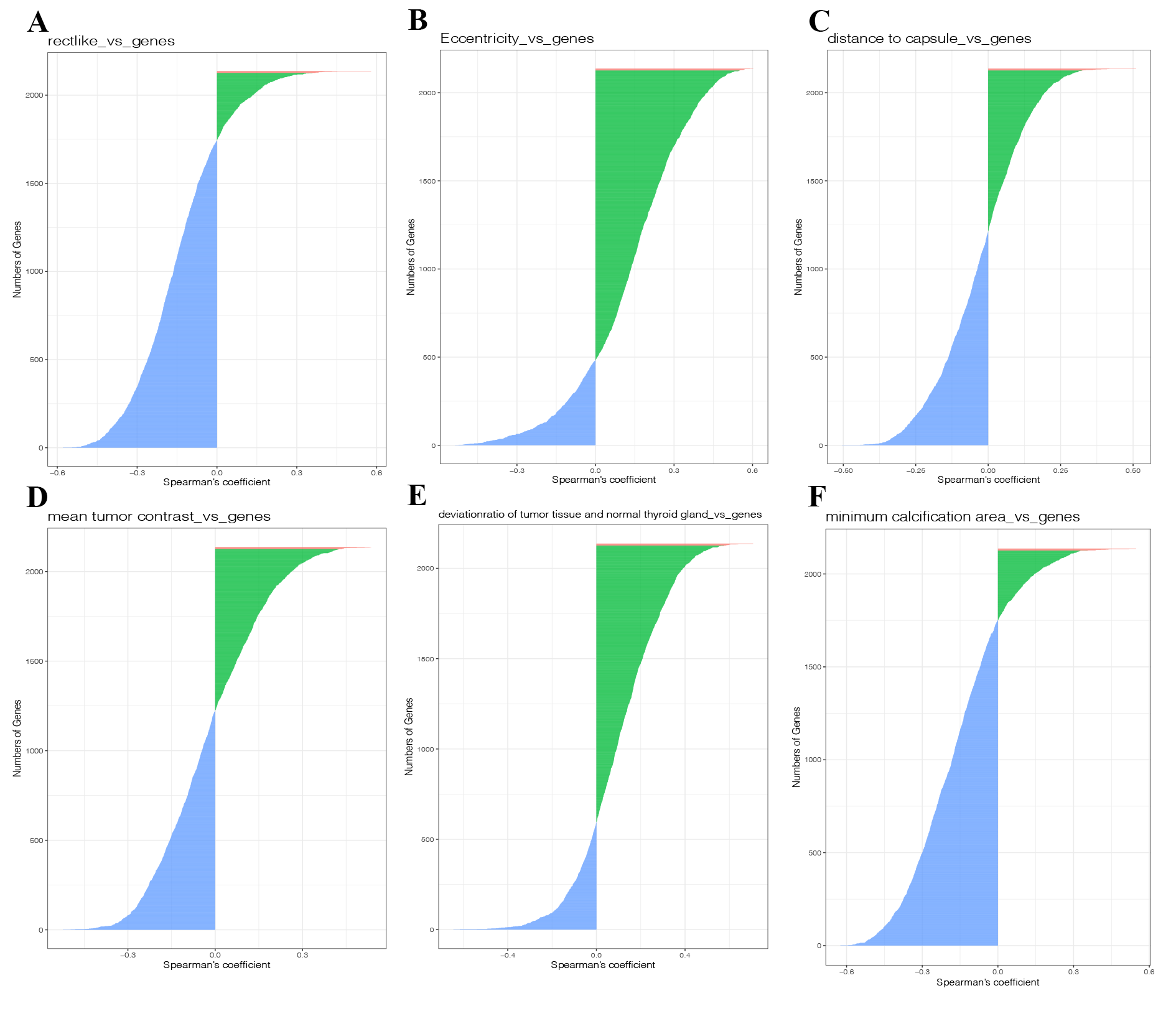


Figure S2. Waterfall plot of Spearman-ranked correlation coefficients for the ranked correlation analysis of radiomics features and normalized gene expression levels. A) rectlike; B) Eccentricity; C) distance to capsule; D) mean tumor contrast; E) deviation ratio of tumor tissue and normal thyroid gland; F) minimum calcification area. Spearman correlation coefficients were sorted from the highest positive (top) to lowest negative (bottom).
